# Isolation and characterization of a novel bacteriophage ST1749 and its effectiveness against *Vibrio parahaemolyticus* and *Salmonella* spp

**DOI:** 10.1016/j.virusres.2025.199579

**Published:** 2025-04-27

**Authors:** Truong Thi Bich Van, Nguyen Thi Loan Anh, Van-Thanh Vo, Nguyen Pham Anh Thi

**Affiliations:** Institute of Food and Biotechnology, Can Tho University, Can Tho City, Vietnam

**Keywords:** Bacteriophage st1749, Bruyoghevirus, Caudoviricetes, Biofilm, Biocontrol, Vibrio parahaemolyticus

## Abstract

Bacteriophages are extensively employed across various fields, including medicine, veterinary medicine, the food industry, agriculture, biotechnology, and pharmacy, owing to their numerous advantages. These advantages include rapid clearance of pathogens, self-propagation at the infection site, host specificity, potential for genetic modification, ease of isolation, stability, and low production costs. This study isolated a bacteriophage from shrimp pond wastewater in the Mekong Delta region. The bacteriophage was identified as a lytic bacteriophage belonging to the genus *Bruyoghevirus*, class *Caudoviricetes*, with the ability to effectively lyse three bacterial strains: *V. parahaemolyticus, Salmonella enteritidis*, and *Salmonella typhimurium*. Growth curve analysis revealed variations in the latency period and the number of phages produced during the life cycle across all three hosts. Bacteriophage Produced 117, 176, and 52 PFU/cell against *V. parahaemolyticus, S. enteritidis*, and *S. typhimurium*, respectively. Phage ST1749 demonstrated activity across a broad range of temperatures (-20 °C to 70 °C) and pH levels (2 to 10), with optimal stability observed at pH 5 to 7. Furthermore, phage ST1749 exhibited biofilm-degrading and lytic capabilities against the three bacterial strains studied. These findings suggest that phage ST1749 has the potential to serve as a biocontrol agent for treating infections caused by antibiotic-resistant bacteria.

## Highlights

• Demonstrates vigorous lytic activity against the genera *Vibrio* and *Salmonella*.
• Significantly reduces biofilm *in vitro* assays.
• Exhibits high stability across various temperatures and pH levels.
• ST1749 produced 117, 176, and 52 PFU per host cell against *V. parahaemolyticus, Salmonella enteritidis*, and *Salmonella typhimurium*, respectively.

## 1 Introduction

Water pollution and waterborne diseases pose a substantial public health concerns, particularly in urban areas where pathogenic bacteria are prevalent (Sharma et al., 2024; Yin et al., 2021). *Vibrio* spp. and *Salmonella* spp. are among the pathogens commonly found in wastewater, leading to considerable environmental and public health challenges.

Pathogenic *Vibrio* species include *Vibrio cholerae, V. parahaemolyticus*, and *V. vulnificus*, with *V. parahaemolyticus* being responsible for severe disease. *This* gram-negative halophilic bacterium is widely distributed in oceans and estuaries (Kim et al., 2017; Raszl et al., 2016). *Vibrio* species are significant pathogens of marine animals and aquaculture populations, and some can cause severe infections in humans through the consumption of contaminated seafood and aquaculture products (Gao et al., 2022; Kang and Chang, 2024; Kim et al., 2017; Kumar et al., 2020; Raszl et al., 2016). Furthermore, *V. parahaemolyticus* can lead to severe acute hepatopancreatic necrosis disease (AHPND) in shrimp aquaculture, resulting in substantial economic losses for shrimp farmers (Kumar et al., 2020). *V. parahaemolyticus* is known for its ability to form biofilms in seafood environments. Unlike *Escherichia coli, Salmonella* spp., and *Listeria monocytogenes, V. parahaemolyticus* employs a dual flagellum system, comprising polar and lateral flagella, to facilitate biofilm formation (Kim and McCarter, 2000). This unique feature enables *V. parahaemolyticus* to navigate diverse conditions, adapt to various environments, and adhere to surfaces. Additionally, *V. parahaemolyticus* produces an active chitinase enzyme, enhancing its adhesion to chitin and copepod surfaces, thereby increasing its ability to infect seafood (Makino et al., 2003). Biofilm communities are encased in extracellular polymeric substances (EPS) and can persist on the surfaces of aquatic plants. The presence of biofilms complicates sanitation efforts and heightens the likelihood of pathogen outbreaks in seafood environments (Wang et al., 2022). The prophylactic and therapeutic use of antibiotics has been extensively employed in hatcheries, transportation, and the sales chain to rapidly control and eliminate the proliferation of pathogenic *Vibrio* species. However, this frequent use has led to the emergence of antibiotic resistance in bacteria (Xu et al., 2016). Increasing research indicates that multidrug-resistant *V. parahaemolyticus* poses a significant threat to public health and economic stability globally (Ghenem and Elhadi, 2017; Kang et al., 2017; Lopatek et al., 2018). In recent decades, antibiotic resistance has become a pressing issue in *V. parahaemolyticus* due to the widespread use of antibiotics in medical and aquaculture settings (Cabello et al., 2013; Elmahdi et al., 2016). Studies consistently report high levels of resistance, particularly to ampicillin, in *V. parahaemolyticus* strains from various sources (Fattel et al., 2019; Jiang et al., 2019; Lee et al., 2018; Mohamad et al., 2019; Ryu et al., 2019). Multidrug resistance is prevalent in this bacterium, complicating antibiotic treatment and management of bacterial infections (Xu et al., 2016). To effectively prevent and control contamination by *V. parahaemolyticus* in aquatic products, it is essential to adopt practices that do not rely on excessive antibiotic use. This approach is a viable solution for economic sustainability and food safety.

*Salmonella* spp., a genus of Gram-negative, rod-shaped bacteria belonging to the Enterobacteriaceae family (Waldman et al., 2020), are responsible for numerous foodborne illnesses globally, with over 93.8 million cases reported annually. These bacteria have emerged as a significant public health concern (de Melo et al., 2021; Mkangara, 2023). *Salmonella* spp. comprises over 2500 distinct serotypes, with more than half belonging to *Salmonella enterica* (Billah and Rahman, 2024). Infections with *Salmonella* spp. are commonly attributed to the consumption of contaminated food or water (Gonçalves-Tenório et al., 2018). *Salmonella* spp. have been detected in various food sources, including meat, vegetables, fresh produce, marine ecosystems, and resident animals, including processed and raw seafood (Carstens et al., 2019; Liu and Kilonzo-Nthenge, 2017; Marin et al., 2022; Maruf Billah and Rahman, 2021). *Salmonella* spp. can form biofilms, facilitating survival in challenging environments and enhance the likelihood of transmission to novel hosts. Biofilms of *Salmonella* spp. contribute to elevated mortality, morbidity, chronic infections, and hospitalization rates due to antibiotic resistance and evasion of the host immune system (Pradhan et al., 2023).

Antibiotic resistance in *Salmonella* spp. poses a growing health concern, particularly due to the emergence of resistance to non-typhoidal *Salmonella* drugs in recent years (Higgins et al., 2020; Marchello et al., 2020). Commonly used antibiotics for *Salmonella* spp. infections include ampicillin, chloramphenicol, and trimethoprim-sulfamethoxazole (Marchello et al., 2020). The extensive use of antibiotics in intensive aquaculture operations has also contributed to the development of antibiotic resistance in *Salmonella* spp. in aquatic ecosystems, seafood, and marine environments (Dewi et al., 2022).

Bacteriophages (phages) are selective viral predators of bacteria. Abundant and ubiquitous, phages can treat bacterial infections (phage therapy), including refractory infections and those resistant to antibiotics (Venturini et al., 2022). Currently, phages are widely used as biological control agents in the food and agriculture industries, wastewater treatment, and aquaculture (D’Accolti et al., 2021). Researchers have reported global efforts to control *Vibrio* spp. and *Salmonella* spp. in wastewater treatment and aquaculture settings using bacteriophages. Previous studies have shown promising results, such as a larval survival rate exceeding 85% in *Penaeus monodon* larvae infected with *V. harveyi* after phage treatment, indicating that phages could be a viable alternative to antibiotics (Karunasagar et al., 2007). Wang et al. (2017) demonstrated the effectiveness of bacteriophage therapy in controlling *V. harveyi* in greenlip abalone (Wang et al., 2017). Le et al. (2020b) showed that lytic bacteriophages could reduce oyster larvae mortality caused by *Vibrio alginolyticus*. Le et al. (2020a) also found that bacteriophages effectively decontaminate *Vibrio* spp. in commercially produced microalgae used as the primary food source for oyster larvae during hatchery culture.

Moreover, the use of bacteriophages in the food industry targeting *Salmonella* spp. is gaining significant attention (Abd-El Wahab et al., 2023; Han et al., 2022; Islam et al., 2023; Khan and Rahman, 2022; Shahdadi et al., 2023; Wessels et al., 2021; Żbikowska et al., 2020). Numerous studies have documented the successful application of commercial *Salmonella* bacteriophages at the laboratory scale (Brenner et al., 2024; Yeh et al., 2017). Other studies have reported promising outcomes, such as the BP phage cocktail exhibiting lytic effects against *Salmonella* spp. isolates (Pourabadeh et al., 2024), a three-phage mixture demonstrating efficacy in controlling *Salmonella* spp. in broth and raw chicken breast (Brenner et al., 2024), and bacteriophage vB_SalS_JNS02 capable of targeting six distinct *Salmonella* serogroups (Li et al., 2024). These viruses are non-toxic to humans, resistant to microbial antibiotic resistance, possess a short research cycle, are low-cost, and allow for easy updates. In the future, bacteriophages could emerge as a promising alternative to antibiotics for combating bacterial pathogens.

This study identified and investigated a novel bacteriophage designated ST1749, capable of infecting multiple hosts. We examined its characteristics, growth behaviour, stability, and host range. Furthermore, we evaluated its potential to inhibit biofilm formation in three bacterial strains: *V. parahaemolyticus, S. enteritidis*, and *S. typhimurium*. Our findings suggest that bacteriophage ST1749 may serve as a valuable tool for controlling infections caused by drug-resistant bacteria.

## 2 Materials and methods

### 2.1 Bacterial cultures and preparations

The bacterial strains used in this study were sourced from the American Type Culture Collection (ATCC) and the National Collection of Type Cultures (NCTC), including *Salmonella enterica* subsp*. enterica serovar Enteritidis* ATCC 49,223, *Salmonella enterica* subsp. *enterica serovar Typhimurium* ATCC 14,028, *Salmonella enterica* subsp. *enterica serovar Enteritidis* ATCC 13,076, *Vibrio parahaemolyticus* ATCC 17,802, *Vibrio parahaemolyticus* NCTC 10,884, *Vibrio parahaemolyticus* NCTC 10,885, *Vibrio vulnificus* NCTC 11,218, *Vibrio vulnificus* ATCC 29,307, *E. coli* ATCC 25,922, and *Pseudomonas aeruginosa* ATCC 27,853. Prior to experimentation, the strains were cultured in Tryptic Soy Broth (TSB; Himedia, India) and Tryptic Soy Agar (TSA; Himedia, India) under optimal conditions, specifically at a temperature of 37 °C with shaking at 120 rpm for 24 hours. This standard procedure ensured the viability and consistent growth of the bacterial strains, which were essential for the reliable and accurate findings of the study.

### 2.2 Isolation and purification of phage

Bacteriophages were isolated from various sources, including slaughterhouse wastewater, pond mud, shrimp pond water, and diseased shrimp samples from the Mekong Delta provinces. The double-layer agar method, as described by Kropinski et al. (2009), was employed for this purpose. Briefly, 2.5 mL of each sample was combined with 7.5 mL of Tryptone Soya Broth (TSB) and incubated overnight at 37 °C with shaking at 120 rpm. Subsequently, the mixture was centrifuged at 16,128 g for 10 min, and the supernatant containing the bacteriophages was collected.

A semi-solid Tryptic Soy Agar (TSA) medium was preheated, combined with bacteria at a 10^6^ CFU/mL concentration, and cultured overnight. Once the bacteria reached the log phase, 5 mL of the mixture was spread on the TSA agar plate. Subsequently, 2 µL of the crude filtrate was added to the solidified semi-solid TSA plate. The plate was then incubated at 37 °C for 24 hours. After incubation, the plates were examined for transparent areas or plaques at the inoculation site.

After incubation, transparent plaques were removed from each plate and placed into a 1.5 mL centrifuge tube containing SM buffer (50 mM Tris–HCl, 100 mM NaCl, 10 mM MgSO_4_ [pH 7.5]). The solution was centrifuged at 16,128 g for 10 min, and the resulting supernatant was collected. The filtered supernatant was titrated using the double-layer plaque assay (Kropinski et al., 2009). After incubating at 37 °C for 24 hours, different plaques were selected based on size and transparency. These plaques were then resuspended in 100 µL of SM buffer, and the purification process continued until phage isolates with homogeneously distributed plaques were obtained. Finally, the purified phage was mixed with 50% (v/v) glycerol and stored at −20 °C until further analysis.

### 2.3 Determination of host range

Ten bacterial strains were utilised in the double-layer plate drop method to determine the host range of the phages. This method involved adding 5 µL of the purified phage culture medium dropwise to the TSA plate containing various bacterial serotypes. Subsequently, the spots were observed and categorised into three groups based on their clarity: transparent, turbid, and unresponsive, after overnight culturing (Shang et al., 2021). To ensure the reliability of the results, the experiment was conducted three times.

### 2.4 Transmission electron microscopy (TEM)

Transmission electron microscopy (TEM) was employed to characterise the morphology of the phage, as outlined in the protocol provided by Taha et al. (2018). A 10 µL sample of the phage, diluted to a concentration of 10^9^ PFU/mL, was stained with 2.5% uranyl acetate for visualization. The sample was subsequently placed on a carbon-coated Cu grid and dried for ten minutes. The sample was then examined using TEM, and images of the stained phage were captured at various magnifications using the advanced high-resolution TEM (1230 JEOL, Tokyo, Japan). The high-quality images obtained facilitated a comprehensive morphological analysis of the phage.

### 2.5 One-step growth curve

A one-step growth curve was generated according to a previous report (Yin et al., 2019), with slight modifications to meet the experiment's needs. To begin with, a culture of bacteria was grown in 5 mL of culture medium at 37 °C with shaking at 120 rpm. Once the culture reached an OD_600_ of 0.6, 1 mL was transferred to a flask containing 4 mL of TSB medium with shaking at 120 rpm. The culture was incubated until the bacterial count reached an OD_600_ of 0.4, which took approximately three hours. At this point, 2 μL of bacteriophage was added to the flask containing the bacterial culture solution. The phage titer was determined every 10 min using the double-layer agar technique. In this technique, a layer of soft agar containing the bacterial culture and phage was poured onto a layer of solid agar. The phage infects the bacterial cells, forming plaques in the bacterial layer. These plaques are then counted to determine the phage titer. Finally, the burst size was calculated as the ratio of the final count of liberated phage particles to the initial count of infected bacterial cells during the latent period. The latent period is the time it takes for the phage to enter the bacterial cell and replicate, forming new phage particles. The burst size is an essential parameter that helps to understand the dynamics of phage infection and the impact of various factors on the phage's lifecycle.

### 2.6 pH and temperature stability

This study examined the thermal and pH stability of isolated and purified phage samples. The samples were cultured at different temperatures (−20 °C, 4 °C, 60 °C, and 70 °C) to assess their resistance to thermal stress. The phage titer was determined at two different time intervals, 30 min and 60 min after culturing, employing the double-layer agar method. Similarly, the pH stability was evaluated by mixing the phage samples with SM buffer adjusted to different pH values (2, 3, 5, 7, 7.5, 9, and 10) using NaOH or HCl solution. After culturing the samples for 2 hours at 37 °C, the phage titer was quantified using the double-layer agar method (Shang et al., 2021) to ascertain the number of surviving phages.

### 2.7 Biofilm inhibition assay

Biofilm reduction was performed using 96-well plates (Danis-Wlodarczyk et al., 2015). Three bacterial strains, *V. parahaemolyticus, S. enteritidis,* and *S. typhimurium,* were cultured overnight in a liquid Tryptic Soy Broth (TSB) medium at 120 rpm. Following this, 100 µL of bacterial suspension was added to each well of the plates and incubated for 24 hours at 32 °C.

After incubation, the bacterial suspension was discarded, and the plates were washed three time with 0.9% NaCl. Subsequently, 100 µL of phage was added to the plates, which were incubated at 32 °C for 4 hours. After this incubation period, the phage and bacteria mixture was removed, and the plates were washed twice with 200 µL of 0.9% NaCl.

Subsequently, 0.1% crystal violet was added to the wells and incubated for 20 min at room temperature. Once the incubation was complete, the crystal violet was removed, and the wells were washed three times with 200 μL of 0.9% NaCl. To dissolve the crystal violet, 30% acetic acid was added to each well. Finally, biofilm formation was evaluated by measuring the optical density at 590 nm (OD_590_ nm) (Coffey and Anderson, 2014).

### 2.8 Lytic activity of phage

An *in vitro* experiment was conducted to study the lytic activity of phages against three strains of bacteria: *V. parahaemolyticus, S. enteritidis,* and *S. typhimurium*. The experiment involved mixing 100 μL of logarithmic-growth phase host bacterial culture containing 10^6^ CFU/mL with an equal volume of phage suspension in a 96-well microplate (Alves et al., 2014). The microplate was then incubated for 0, 2, 4, 6, and 24 hours. At each time point, the optical density of the mixture was measured at OD_600_ nm. This entire procedure was repeated three times to ensure reliable and consistent results.

## 3 Results

### 3.1 Isolation and morphological characteristics of phage ST1749

**Fig. 1:**
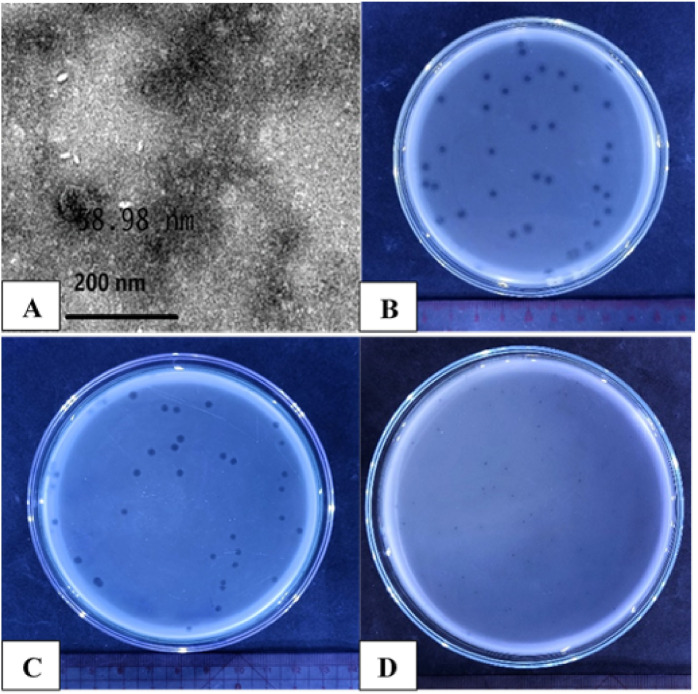
Transmission electron microscopy images **(A)** and plaque morphology **(B, C, D)** of phage ST1749.

This research tested, the lytic activity of phage ST1749 against three bacterial strains, *V. parahaemolyticus* ATCC 17,802, *S. enteritidis* ATCC 49,223, and *S. typhimurium* ATCC 14,028, was tested after isolation from shrimp pond wastewater. Phage ST1749 morphology on TSA plates is characterised by small, transparent, round plaques with a halo region at the plaque periphery on the host *V. parahaemolyticus* (Fig. 1B). The plaques on *S. enteritidis* and *S. typhimurium* are also round and transparent but lack halos at the periphery. (Fig. 1C, D). The purified ST1749 particles were analysed through TEM, revealing a polyhedral head with a diameter of 58.98 nm (Fig. 1A). According to the International Committee on Taxonomy of Viruses guidelines (Turner et al., 2023), phage ST1749 is classified as a member of the *Bruynoghevirus* genus in the class *Caudoviricetes* based on its morphological characteristics.

**(A)** Transmission electron microscopy images of phage ST1749 were captured using the state-of-the-art TEM (JEOL 1230, Tokyo, Japan); **(B)** Plaque morphology of phage on *V. parahaemolyticus*; **(C)** Plaque morphology of phage on *S. enteritidis*; **(D)** Plaque morphology of phage on *S. typhimurium.*

### 3.2 Host specificity

The host range of the phage was assessed against *V. parahaemolyticus* ATCC 17,802 using the double-layer plate drop method. The results indicated that phage ST1749 forms distinct plaques against the host bacteria, demonstrating robust lytic activity. Furthermore, it exhibited susceptibility to *Salmonella enterica* subsp. *enterica serovar Enteritidis* ATCC 49,223 and *Salmonella enterica* subsp. *enterica serovar Typhimurium* ATCC 14,028 against the tested strains. It failed to form plaques against other bacterial strains (Table 1).

**Table 1:** Host range of the isolated phage ST1749 against ten bacterial strains.

Bacterial strains	Phage sensitivity
*Salmonella enterica* subsp*. enterica serovar Enteritidis* ATCC 49,223	+
*Salmonella enterica* subsp. *enterica serovar Typhimurium* ATCC 14,028	+
*Salmonella enterica* subsp. *enterica serovar Enteritidis* ATCC 13,076	–
*Vibrio parahaemolyticus* ATCC 17,802	+
*Vibrio parahaemolyticus* NCTC 10,884	+
*Vibrio parahaemolyticus* NCTC 10,885	–
*Vibrio vulnificus* NCTC 11,218	–
*Vibrio vulnificus* ATCC 29,307	–
*E. coli* ATCC 25,922	+
*Pseudomonas aeruginosa* ATCC 27,853	–

### 3.3 One step-growth curve

**Fig. 2:**
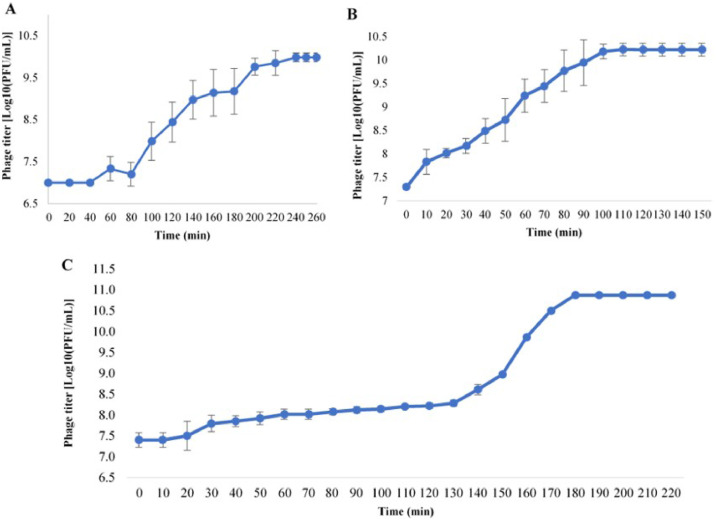
One-step growth curve of phage ST1749.

The study revealed that bacteriophage development exhibited distinct patterns across the three host species. For *V. parahaemolyticus*, the phage exhibited a latent period of 80 min, maintained stability for 200 min, and had a burst size of 117 PFU per infected cell (Fig. 2A). In contrast, for *S. enteritidis*, the latent period was 10 min, stability at 100 min, and a burst size of 176 PFU/infected cell (Fig. 2B). Lastly, for *S. typhimurium*, the latent period was 30 min, stability at 180 min, and a burst size of 52 PFU/infected cell (Fig. 2C). Notably, phage ST1749 demonstrated a short latent period and high burst size on *S. enteritidis*.

**(A)** On host *V. parahaemolyticus*; **(B)** On host *S. enteritidis*; **(C)** On host *S. typhimurium.*

### 3.4 pH and temperature stability

**Fig. 3:**
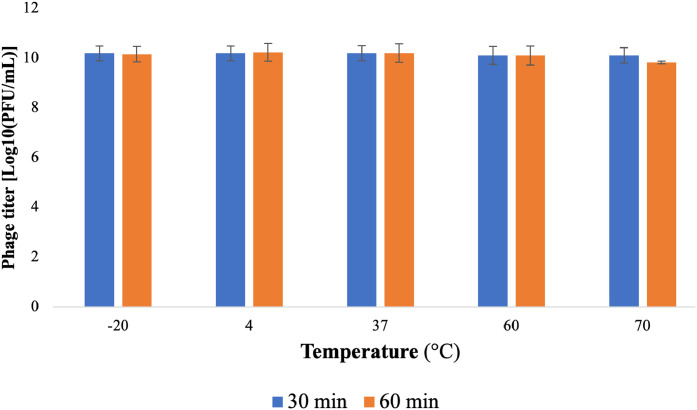
Thermal stability of phage ST1749.

**Fig. 4:**
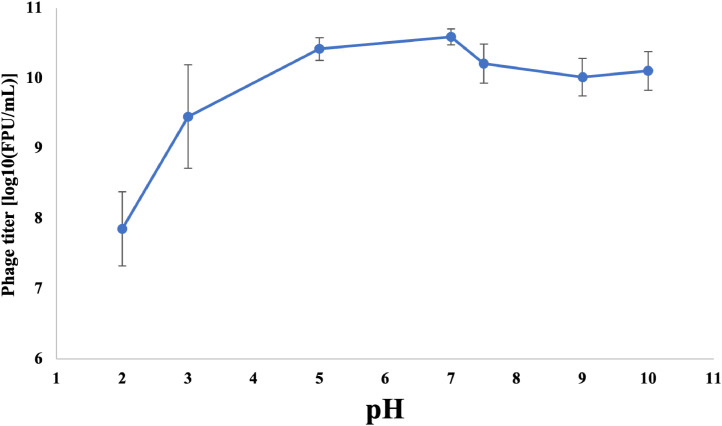
pH stability of phage ST1749.

Phage ST1749 has been observed to retain its infectious capacity even after incubation at extreme temperatures of −20 °C or 60 °C for 30 and 60 min, respectively. Notably, it retains its operational capacity at 70 °C (Fig. 3). Additionally, the stability of the isolated and purified phage ST1749 was assessed at various pH levels, including 2, 3, 5, 7, 9, and 10 (with pH=7.5 serving as the control). The experimental results demonstrated that phage ST1749 remained active under pH conditions from 2 to 10, with optimal activity observed within the pH range of 5 to 7 (Fig. 4).

### 3.5 Biofilm inhibition assay

**Fig. 5:**
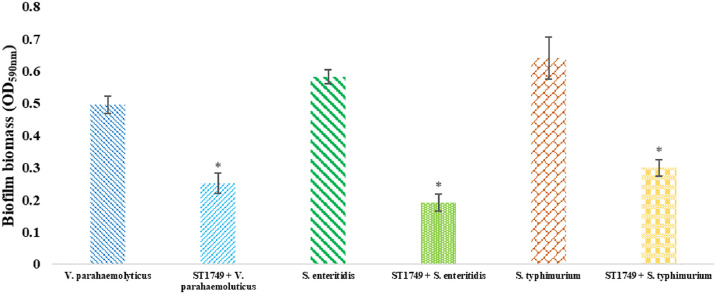
Effect of phage ST1749 on inhibition of biofilm in 96-well microplate after 24 hours. Values represent mean with a standard deviation of three determinations. * Significant at *p* < 0.01.

After a four-hour treatment with phage ST1749, the biofilm of three bacterial strains (*S. enteritidis, S. typhimurium*, and *V. parahaemolyticus*) exhibited a substantial reduction, as evidenced in Fig. 5. The optical density (OD) results demonstrated a decrease in the biofilm values of the bacterial strains *S. enteritidis, S. typhimurium*, and *V. parahaemolyticus* by 0.192, 0.299, and 0.252, respectively. These values were significantly lower compared to the control group without treatment, which exhibited OD values of 0.582, 0.641, and 0.496, respectively. The statistical significance of these results was determined using a significance level of *p* < 0.01.

### 3.6 Lytic activity of phage

**Fig. 6:**
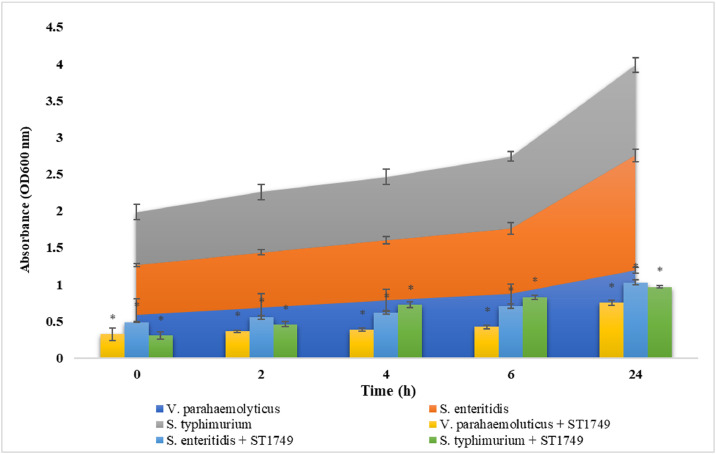
Lytic activity of phage ST1749 at five time intervals 0, 2, 4, 6, and 24 hours. Values represent mean with a standard deviation of three determinations. * Significant at *p* < 0.01.

Bacteriophage lytic activity quantifies the capacity of bacteriophages to lyse host bacterial strains. This study, based on the bacteriophage’s life cycle, assessed the lytic ability of bacteriophages at five time intervals: 0, 2, 4, 6, and 24 hours. Optical density (OD) measurements indicated that bacteriophage treatments significantly reduced the OD value compared to the untreated samples at all five time points. Fig. 6 demonstrates that phage ST1749 exhibited the highest lytic ability against *V. parahaemolyticus*. At 0 hours, the OD value of the phage-treated sample was 0.32, substantially lower than the untreated sample’s value of 0.59. From 2 to 6 hours, the OD value of the phage-treated sample decreased steadily, while at 24 hours, it sharply dropped from 1.19 to 0.75. Phage ST1749 also demonstrated significant lytic ability against *S. enteritidis* and *S. typhimurium*, decreasing OD values during the corresponding treatment time intervals.

## 4 Discussion

Phage therapy offers advantages over conventional bacterial control methods. Phages, the most abundant and genetically diverse organisms on Earth, facilitate easy detection. They exhibit high specificity and narrow host targeting, significantly reducing adverse effects on the ecosystem. Furthermore, phage therapy utilises self-replicating phages, enabling the administration of small yet effective doses. Bacteriophages possess anti-biofilm properties, allowing them to penetrate biofilms and effectively eliminate bacteria. Additionally, they are limited by becoming prey to macrophages after eliminating host bacteria. Notably, bacteriophages can evolve to overcome both bacteriophage resistance and antibiotic resistance (Thakali et al., 2024). Bacteriophages have a history of being beneficial in wastewater treatment systems and controlling antibiotic-resistant bacteria. The potential application of lytic phages to control concentrations of *V. parahaemolyticus, S. enteritidis, S. typhimurium, E. coli*, and gas-producing bacteria has been discussed in previous studies (Azzam et al., 2024; Hsu et al., 2024; Khan et al., 2024; Kim et al., 2024; Kim et al., 2024; Liu et al., 2024; Liu et al., 2024; Martinez-Soto et al., 2024; Sun et al., 2024; Taj et al., 2024; Torkashvand et al., 2024; Van et al., 2023; Van and Thu, 2023; Withey et al., 2005; Zhang et al., 2024). A crucial step in developing effective phage therapy involves generating a well-defined and meticulously curated library of lytic phages, encompassing host range studies and their bactericidal activity.

This study identified a new phage capable of effectively controlling the bacteria *V. parahaemolyticus, S. enteritidis*, and *S. typhimurium*. This achievement is attributed to the phage’s ability to lyse, control biofilms, and exhibit characteristics of bacteriophages. The appearance of plaques formed by the ST1749 phage varied depending on the host, underscoring the significance of host specificity in phage behaviour. Plaques were created by spreading the phage and host bacteria on a solid agar medium to restrict phage diffusion (Kropinski et al., 2009). After incubation at 37 °C for three hours, plaques were observed on the bacterial layer of the semi-solid medium. These plaques are initiated by a single phage virion or a phage-infected bacterium and spread spherically to form circular plaques characterised by reduced turbidity and decreased opacity compared to the bacterial matrix. The development process of phage plaques can vary depending on the host type, phage type, and adhesion conditions, making them easily observable in laboratory settings. Plaques serve as a widely used method for observing phage activity, and macroscopic techniques are employed for isolating and purifying phage strains, followed by their classification. Furthermore, the morphology of phage plaques is correlated with the one-step growth curve, adsorption rate, lysis time of bacteriophages (Abedon, 2021), and their diffusion rate within a specific environment (Abedon and Yin, 2009).

In addition to the distinct plaque morphology, this study also observed a notable disparity in the one-step growth curve of phage ST1749. Notably, on *S. typhimurium*, the plaques exhibit reduced size and produce fewer bacteriophages than those observed on *S. enteritidis*. However, establishing a direct correlation between the varying plaque phenotypes and the life cycle of ST1749 on *V. parahaemolyticus* and *S. enteritidis* remains challenging. The study revealed that phage ST1749 exhibits a relatively short latent period on *S. enteritidis* and *S. typhimurium*. Conversely, on *V. parahaemolyticus*, the latent period is significantly extended, spanning approximately 80 min. The latent period of bacteriophages refers to the duration between their entry into the host bacterium and the subsequent lysis and release of progeny. At the same time, burst size indicates the number of newly released bacteriophages by a single host bacterium. The incubation time and the number of phages formed after one life cycle of bacteriophages are crucial factors in their ability to combat bacteria (Yang et al., 2020). A shorter latent period indicates a stronger ability to kill bacteria (Li et al., 2020), which holds true for *S. enteritidis* and *S. typhimurium*. In the case of *V. parahaemolyticus*, although the latent period is longer, the burst size of phages in one lifetime is relatively large and consistent, with 117 PFU/host cell.

Bacteriophages with anti-biofilm properties are highly sought after due to their ability to penetrate biological membranes and effectively eliminate bacteria. A biofilm is a structure composed of bacteria and other microorganisms embedded in an extracellular matrix of organic substances produced by these microorganisms. Biofilms are more challenging to remove than planktonic forms due to the protective properties of the matrix (Augustyniak et al., 2021). This protection enables pathogens, antibiotics, disinfectants, and other chemicals to evade the immune system (Jamal et al., 2018; Roy et al., 2018). In this study, phage ST1749 effectively disintegrated biofilms of *S. enteritidis, S. typhimurium*, and *V. parahaemolyticus* after only four hours of treatment. Lytic bacteriophages can be an effective weapon in the battle against biofilms, preventing and eliminating their formation. Numerous attempts to utilise bacteriophages as preventive measures have been documented (Melo et al., 2016; Santiago and Donlan, 2020). One advantage of bacteriophages is that they operate differently from antibiotics, producing enzymes such as depolymerase that can destroy biofilm matrices composed of polysaccharides, including exopolysaccharides (EPS) or alginase (Santiago and Donlan, 2020).

Furthermore, bacteriophages can induce host bacteria to synthesise enzymes and proteases that degrade bacterial EPS. In contrast to antibiotics, bacteriophages can specifically target bacteria with diminished metabolic activity due to nutrient scarcity. They can penetrate biofilms through water channels via diffusion or absorption by motile bacteria, thereby augmenting their efficacy in eliminating biofilms (Amankwah et al., 2021; Atshan et al., 2023; Geredew Kifelew et al., 2019). These mechanisms facilitate phage entry, replication, and the accumulation of increased bacterial density within the biofilm, releasing novel virions. Even when targeting cells with reduced metabolic activity, lytic phages demonstrate effectiveness by releasing intracellular materials that stimulate bacterial metabolism and ensure enduring effects (Amankwah et al., 2021). When exposed to a wide host range, multihost bacteriophages emerge as valuable assets in disrupting polymicrobial biofilms.

The temperature and pH tolerance of bacteriophages are crucial for biocontrol applications. To study the viability of phage ST1749 under various environmental conditions, the temperature and pH stability of the phage were determined by assessing its plaque-forming units (PFU). The results indicated that phage ST1749 exhibits a broad range of tolerance to temperature and pH levels. This finding aligns with previous studies suggesting that bacteriophages are more resistant to alkaline conditions than acidic ones (El-Dougdoug et al., 2019; Jiang et al., 2021).

Some related reports indicate that phage vB_SalP_LDW16 is stable at temperatures between 40 and 60 °C and decreases above this temperature. The titer of phage vB_SalP_LDW16 decreases significantly when pH is less than 5 and becomes inactive at pH 2 (Cao et al., 2022). Phage KSL-1 is sensitive to temperatures above 80 °C and stable at a wide alkaline pH range (Sun et al., 2012). Phage swi3 is stable at temperatures below 50 °C and in the pH range of 6 to 8 (Sui et al., 2021). Phage HY01 is stable in the temperature range below 65 °C and the pH range of 4 to 11 (Lee et al., 2016). Phage VP06 is inactivated at 60 °C and stable at pH 7 to 11 but is inactivated at pH 2 to 3 (Wong et al., 2019).

The physiological stability of bacteriophages is essential, as pH and temperature conditions can impact their stability and bactericidal activity (Duc et al., 2020). Understanding the stability and activity of bacteriophages is crucial for determining their applications. Additionally, the ability of any antibacterial agent to withstand acidic pH conditions is a significant requirement. Bacteriophages can be stored at temperatures below 0 °C for a short period. However, previous studies showed a decrease in phage titer at temperatures below the freezing point due to crystal formation. Therefore, it is recommended to use glycerol (Gwak et al., 2018). Further studies are necessary to assess the stability of frozen phages over extended periods. These findings can serve as a guide for developing strategies to control phage infections through heat treatment of equipment and the implementation of effective cleaning practices (Pringsulaka et al., 2011).

To elucidate these complexities, a comprehensive analysis of the genome sequence of phage ST1749 is imperative to clarify its intricate mechanisms of action. This analysis will be presented in a forthcoming research publication. The complete genome sequence of the ST1749 phage has been deposited in GenBank under accession number PQ390404.1.

## 5 Conclusion

This study identified a bacteriophage, ST1749, that effectively lysed three bacterial strains: *V. parahaemolyticus, S. enteritidis*, and *S. typhimurium*. Phage ST1749 demonstrated robust anti-biofilm activity and lytic properties against the tested bacterial strains, indicating its potential as an antibacterial agent for combating infections caused by antibiotic-resistant bacteria.

## Funding

This work was supported by the Vietnam National Foundation for Science and Technology Development (NAFOSTED) under grant number: 106.04–2019.335.

## Ethical approval

Not applicable.

## CRediT authorship contribution statement

**Truong Thi Bich Van:** Writing – review & editing, Writing – original draft, Supervision, Resources, Project administration, Methodology, Investigation, Funding acquisition, Formal analysis, Data curation, Conceptualization. **Nguyen Thi Loan Anh:** Writing – original draft, Resources, Methodology, Formal analysis, Data curation. **Van-Thanh Vo:** Software, Methodology, Formal analysis, Data curation. **Nguyen Pham Anh Thi:** Software, Formal analysis, Data curation.

## Declaration of competing interest

The authors declare that they have no known competing financial interests or personal relationships that could have appeared to influence the work reported in this paper.

## Data Availability

The datasets generated during and analyzed during the current study are available from the corresponding author upon reasonable request.
